# Nurses’ supervision and autonomy in hospitals: an Ibero-American multicenter study

**DOI:** 10.1590/0034-7167-2025-0278

**Published:** 2026-07-27

**Authors:** Rebecca Maria Oliveira de Góis, Gilberto Tadeu Reis da Silva, Maria Lúcia Silva Servo, Thadeu Borges Souza Santos, Edenise Maria Santos da Silva Batalha, Monica Motta Lino, Silvana Lima Vieira, Deybson Borba de Almeida

**Affiliations:** I Universidade Federal da Paraíba. João Pessoa, Paraíba, Brazil; II Universidade Federal da Bahia. Salvador, Bahia, Brazil; III Universidade Estadual de Feira de Santana. Feira de Santana, Bahia, Brazil; IV Universidade Estadual da Bahia. Salvador, Bahia, Brazil; V Universidade Federal de Santa Catarina. Florianópolis, Santa Catarina, Brazil

**Keywords:** Nursing, Nursing, Supervisory, Professional Autonomy, Hospitals, Multicenter Study., Enfermería, Supervisión de Enfermería, Autonomía Profesional, Hospitales, Estudio Multicéntrico.

## Abstract

**Objectives:**

to analyze nursing supervision contributions and influences in the construction and implementation of regulated professional autonomy of nursing teams in hospital settings in Ibero-American countries.

**Methods:**

qualitative research, stemming from a multicenter study conducted in university hospitals in Portugal, Spain, and Brazil. Semi-structured interviews were conducted with clinical nurses and ward managers. Content analysis was used to interpret the collected data.

**Results:**

supervision and its potential for developing autonomy were influenced by institutional hierarchy, the structure of decision-making processes, and training processes. A supervision approach based on support and professional development favors nurses’ autonomy, while practices centered on control can limit it.

**Final Considerations:**

the emphasis on supervision models that promote nurses’ development and participation contributes to more autonomous and effective care practices.

## RESUMEN

**Objetivos:**

analizar las contribuciones e influencias de la supervisión enfermera en la construcción e implementación de la autonomía profesional regulada de los equipos de enfermería en entornos hospitalarios de países iberoamericanos.

**Métodos:**

investigación cualitativa, derivada de un estudio multicéntrico, realizada en hospitales universitarios de Portugal, España y Brasil. Se realizaron entrevistas semiestructuradas con enfermeras clínicas y gestores de pacientes hospitalizados. Se utilizó el análisis de contenido para interpretar los datos recopilados.

**Resultados:**

la supervisión y su potencial para desarrollar la autonomía se vieron influenciados por la jerarquía institucional, la estructura de los procesos de toma de decisiones y los procesos de formación. Un enfoque de supervisión basado en el apoyo y el desarrollo profesional favorece la autonomía de las enfermeras, mientras que las prácticas centradas en el control pueden limitarla.

**Consideraciones Finales:**

el énfasis en modelos de supervisión que promueven el desarrollo y la participación de las enfermeras contribuye a prácticas de atención más autónomas y eficaces.

## INTRODUCTION

Supervision is intrinsically linked to nurses’ work process. It is noted that supervisory practice in hospital settings interfaces with the management model adopted to achieve strategic objectives and goals, as well as to leverage quality and accreditation standards in healthcare services, in addition to impacting professionals’ performance^(1)^. In this context, supervision is of fundamental importance in the autonomous decision-making of nurses and in the provision of safe care. In addition to fostering team integration and motivation, it contributes to the production of more efficient and humanized care in hospital settings^(2,3)^.

The supervision process, along with planning and assessment, is essential for managing healthcare services, aligning the organization’s mission, vision, values, and business with hospital workers’ daily activities. Team monitoring and follow-up techniques, such as constructive feedback, meetings, 360-degree performance assessments, case analysis, and remote supervision, built on group and individual techniques, have been widely used to optimize supervision and promote improvements in healthcare. It is worth noting that the main instruments used in the nursing supervision process are medical records, care plans, theory-driven nursing process (NP), supervision plans, work schedules, establishment of monitoring and assessment indicators, the Deming method for quality improvement, and health/nursing service flows, care guidelines, and manuals/regulations with instructions on standards and routines^(4,5)^.

It is noted that the performance of the professionals who perform this function requires the mobilization of a set of skills and abilities that involve specific technical, administrative, and political knowledge and the ability to implement the performance management of the organization and the people on the nursing team, thus contributing to professional autonomy construction/strengthening^(6,7)^.

In this regard, it is important to state that autonomy is a characteristic that is always regulated in teamwork, as the autonomy of each and every professional is relative to the political, legal and institutional framework, namely clinical protocols, therapeutic guidelines, health laws, regulations of professional councils, and Brazilian Health System policies.

However, supervision is essential for strengthening professional autonomy, even when regulated, understood as nurses’ ability to make independent decisions within an established tacit system and effectively exercise their responsibilities. Studies show that structured supervisory practices improve trust, problem-solving skills, and technical competence among professionals, enabling not only improved quality of care but also the development of their performance as leaders and care organizers^(8,9)^. Furthermore, interactive approaches, such as the use of active methodologies and advanced technologies, have shown promising results in training nurses for complex contexts^(10)^.

Nurse autonomy development and implementation are also related to the strengthening of knowledge and skills throughout the training process. Reflections on nurses’ identity and work processes, as well as the meaning of autonomy, should be encouraged from the undergraduate level, promoting more effective professional practice focused on shared decision-making^(9-11)^. From this perspective, supervision is seen as a pedagogical device that not only provides technical guidance, but also contributes to managing team members’ personal and professional performance^(12)^. Additionally, recent studies reinforce that supervision, when carried out in a structured manner, plays a crucial role in people management policies and in the development of resilient and respectful work settings^(13)^.

Based on the above, this study seeks to analyze nursing supervision contributions and implications in regulated professional autonomy construction and implementation in hospital settings in Ibero-American countries. By addressing different historical, cultural, social, and political contexts of nursing, this work aims to reflect on the strengths, gaps, and challenges of this nursing supervision process, fostering care practices that align with more equitable, efficient, and effective models.

### Study relevance

This article is a product of a thesis^(14)^ entitled “*Processo de supervisão do enfermeiro: contributos para o desenvolvimento da autonomia no ambiente hospitalar*”, which is deposited in the institutional repository through the link: https://repositorio.ufba.br/handle/ri/37325.

## OBJECTIVES

To analyze nursing supervision contributions and intersections in the construction and implementation of regulated professional autonomy of nursing teams in hospital settings in Ibero-American countries.

## METHODS

### Ethical aspects

This research was approved by the Research Ethics Committee and is linked to the matrix project “*Modelos de Gestão Hospitalar em Enfermagem: memórias de enfermeiros*”. All participants signed the Informed Consent Form, guaranteeing their voluntary, autonomous, conscious, free, and informed participation. The ethical principles of Law 14,874/2024 and international ethical standards were respected. To preserve their identity, pseudonyms were assigned to participants, starting with the letter “N” for nurse, followed by “Br” for Brazil, “Sp” for Spain, and “Po” for Portugal, in addition to a number.

### Study design

This was a multicenter, qualitative, descriptive, and exploratory study conducted in three Ibero-American countries (Brazil, Portugal, and Spain). The COnsolidated criteria for REporting Qualitative research guidelines were followed, reinforcing methodological rigor for qualitative research.

### Methodological procedures

The guiding questions were developed based on a semi-structured questionnaire containing characterization data such as time since graduation and professional qualifications. It is worth noting that this study focused on deepening the discussion to analyze nursing supervision’s contribution to building/strengthening professional autonomy in hospital settings in Ibero-American countries.

### Study setting

The study was conducted at university hospitals located in Bahia, Brazil, Coimbra, Portugal, and Toledo, Spain. These institutions were chosen because they represent teaching, research, and healthcare settings, as well as because they reflect different nursing and healthcare management models.

### Data source

The study included 30 nurses, nine Brazilians, eight Spaniards, and 13 Portuguese. Nurses who currently work or have worked as top-level managers, middle managers, or unit/service managers with at least six months of experience in the role were included. Nurses on vacation, maternity leave, or sick leave during the data collection period, as well as those who did not respond after three attempts to schedule an appointment or who refused to participate, were excluded.

### Data collection and organization

Data collection was conducted through semi-structured interviews between September 2020 and February 2021 in private rooms at the hospitals. The interviews were based on a previously validated script consisting of five guiding questions and addressed sociodemographic aspects, organizational management models, and supervisory practices. The questions explored topics such as decision-making, performance assessment, management action planning, and educational processes. The interviews, which lasted an average of 60 to 120 minutes, were recorded and transcribed. Participant selection used the snowball sampling technique, in which nurses recommended other potential participants based on their referral networks. After transcription for validation of interviews with participants, data saturation was reached, and it was determined that data collection needed to be terminated as no new aspects emerged in the list of responses.

### Data analysis

Thematic content analysis involved the following stages: (i) initial exploration: understanding the sociohistorical context of the analyzed social groups; (ii) data organization: systematizing the collected information and organizing the statements into meaning clusters; and (iii) data classification: constructing inferences and interpretations based on the study’s theoretical foundations. NVivo^®^ 11 and webQDA^®^ software were used to organize the data and facilitate qualitative analysis. Thematic analysis identified one main category: “Potentials related to the contribution of supervision to the development/strengthening of nurses’ autonomy in hospital settings”.

## RESULTS

Chart 1 highlights characteristics of participants by age, sex, marital status, race, and religion. Females, married people, and Catholics were more prevalent in all three countries. Concerning age, in Portugal, the majority were between 51 and 60 years old, while in Brazil and Spain, they were between 30 and 40 years old.

**Chart 1:** Characterization of sociodemographic information of subjects interviewed in Brazil, Portugal and Spain (2020) at the *Hospital Universitário Professor Edgard Santos* (Salvador, Bahia, Brazil), *Universidade de Coimbra* hospitals (Coimbra, Portugal) and the University Hospital Complex of Toledo (Toledo, Spain).

SOCIODEMOGRAPHIC INFORMATION		PORTUGAL	SPAIN	BRAZIL
**Age**	30 to 40 years old	2	3	4
	Sex	1	0	2
	Marital status	7	2	2
	Skin color	3	1	1
	Religion	0	2	0
**Sex**	Female	9	7	8
	Male	4	1	1
**Marital status**	Single	2	1	2
	Married	9	4	6
	Divorced	1	0	1
	Widowed	1	0	0
	Not provided	0	3	0
**Skin color**	White	7	0	2
	Black	0	0	2
	Brown	0	0	5
	Caucasian	1	0	0
	Not provided	5	8	0
**Religion**	Catholic	9	3	5
	Christian	0	0	1
	Evangelical	0	0	1
	Spiritualist	0	0	1
	No religion	0	0	1
	Protestant	2	0	0
	Atheist	1	0	0
	Not provided	1	5	0

Furthermore, participants’ characteristics are presented according to their level of education and length of training in Brazil, Portugal, and Spain. Regarding education level in the three settings investigated, there is a discrepancy between countries, as shown in Chart 2.

**Chart 2:** Characterization of academic information of subjects interviewed in Brazil, Portugal, and Spain (2020) at the *Hospital Universitário Professor Edgard Santos* (Salvador, Bahia, Brazil), *Universidade de Coimbra* hospitals (Coimbra, Portugal) and the University Hospital Complex of Toledo (Coimbra, Portugal), and the University Hospital Complex of Toledo (Toledo, Spain)

ACADEMIC INFORMATION		PORTUGAL	SPAIN	BRAZIL
**Education**	University degree with graduate studies/specialization	5	3	5
	University degree in nursing	1	0	0
	Graduate degree with a master’s degree	7	2	1
	Graduate degree with a doctoral degree	0	1	2
	Postdoctoral degree	0	0	1
	Not provided	0	2	0
**Length of training**	5 to 10 years	0	0	1
	11 to 20 years	2	3	4
	21 to 30 years	2	2	2
	31 to 37 years	6	3	2
	38 to 40 years	1	0	0
	41 to 50 years	2	0	0
**Higher education institution**	*Universidade Federal da Bahia (public)*	0	0	5
	*Universidade Federal do Rio Grande do Sul (public)*	0	0	1
	*Universidade Católica de Salvador (privada)*	0	0	2
	*Universidade Jorge Amado (private)*	0	0	1
	*Escola Superior de Enfermagem Dr. Ângelo da Fonseca*	6	0	0
	*Escola Superior de Enfermagem de Coimbra*	1	0	0
	*Escola Superior de Enfermagem de Sá Barreto*	2	0	0
	*Escola Superior de Enfermagem de Bissaya Barreto*	4	0	0
	*Distance university*	0	1	0
	*Universidad de Castilla-La Mancha*	0	1	0
	*Universidade Complutense de Madrid*	0	1	0
	*Universidad Rovira i Virgili*	0	1	0
	*Universidad Autónoma de Madrid*	0	1	0
	*Not informed*	0	3	0

**Figure 1:**
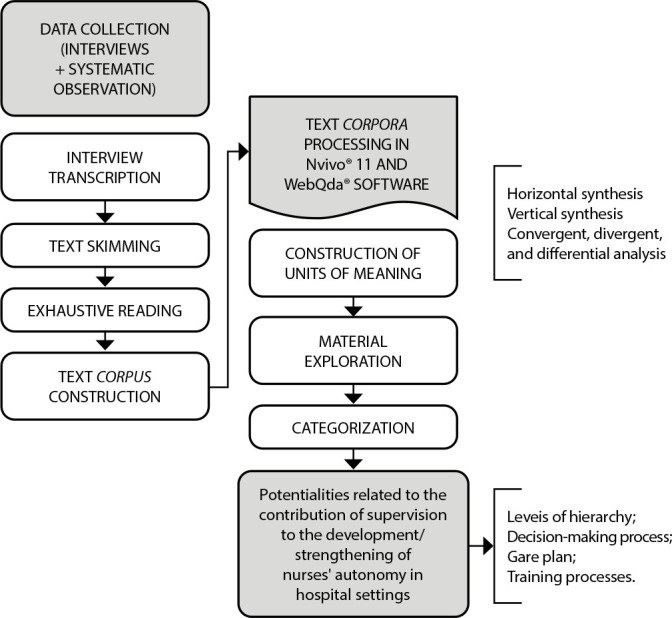
Summary of the category regarding nursing supervision contribution to professional autonomy construction/strengthening in hospital settings in Ibero-American countries (Salvador, Bahia, Brazil; Coimbra, Portugal; Toledo, Spain)

Furthermore, Chart 2 characterizes the participants according to education level, length of training, and training institution. Regarding education level in the three settings investigated, there is a discrepancy between countries, with the majority in Portugal holding a master’s degree, and in Spain and Brazil, a specialist degree. Concerning length of training, in Portugal, the number of participants in Spain and Brazil exceeded 30 years, which differs from participants in Spain and Brazil. Figure 1 summarizes the data collection process leading up to the development of the study categories.

### Potentialities related to supervision contribution to the development/strengthening of nurses’ autonomy in hospital settings

Nurses’ autonomy in hospital settings is influenced by several factors, including the management and healthcare model, often represented by the hierarchical structure of supervision within the organization. The statements of participants from Portugal, Spain, and Brazil reflect different perspectives on how this hierarchy affects their professional autonomy.

In the Brazilian context, supervision supports unit leaders through figures such as “reference nurse”, providing direct support to teams. One of the Brazilian nurses highlights the autonomy granted by intermediate managers, which allows professionals to manage their demands and team, with management offering support and maintaining open communication.

[...] *this autonomy within each unit, this support from the division, we have a person who is responsible for this supervision, so it is as if today the person who is responsible, right, for supporting these unit heads, is actually called today a reference nurse.* (NBr-03)

*I’m living what I’ve always lived, which I think is right* [...] *a leadership that gives you complete autonomy, you, the manager, you who will know how to act, who knows your demands,* [...] *your team,* [...] *you will do your best, I just want your feedback.* [...] *a management team that supports us a lot, that listens to us a lot. So, today I can go to the division at any time. I’m always very welcome, the door is always open, there’s no “oh, don’t come now because I’m super busy”. I feel much more welcomed, and today I have much more autonomy, so this has reflected much more directly in my work and in my team.* (NBr-06)

In Spain, autonomy is perceived at the professional level, allowing nurses to make decisions within established ethical and practical parameters. A Spanish nurse mentions that, although autonomy exists, it is conditional on compliance with ethical standards and institutional practices.

*Yes, there is autonomy, but at a professional level we would say, for your direct work, if there is any other, as long as you don’t do anything that is unethical or wrong, it is praxis, but if there is a certain autonomy, there is a certain autonomy.* (NSp-05)

In Portugal, supervision is monitored at the operational level, with head nurses and service directors responsible for specific indicators. Portuguese professionals emphasize the importance of resolving issues internally, resorting to hierarchy only when necessary, demonstrating a collaborative approach focused on problem-solving within teams.

*There is monitoring at the operational level, that is, the head nurse, the service director, is responsible for the indicators contracted for their service.* (NPo-02)

*We only transfer to the hierarchy everything that we cannot actually resolve internally or that we believe we can make an important contribution to a more sustained decision.* (NPo-04)

*It depends on the problem. Normally, I don’t ask my supervisor questions unless it’s something that involves the institution, a serious complaint, a serious incident, or something that obviously requires their knowledge. However, for day-to-day issues, I only ask if I can’t resolve them myself.* (NPo-10)

These different perspectives highlight how the hierarchical structure and organizational culture in each country influence nurses’ autonomy in hospital settings. Understanding these nuances is essential to improving supervision practices that strengthen professional autonomy.

Nurses’ decision-making in hospital settings is shaped by several factors, including organizational structure, professional autonomy, and leadership practices. The experiences shared by nurses from Brazil, Spain, and Portugal offer a comprehensive overview of how these factors influence their daily decisions. However, it is clear that this process is contingent, at times unsystematic, and monocratic.

In Brazil, nurses emphasize the importance of institutional strategic planning, which involves identifying strengths and weaknesses, analyzing internal and external settings, and defining goals and indicators. Furthermore, they emphasize autonomy in managing their teams and the need for personal development, such as participating in coaching sessions, to strengthen decision-making and effective leadership.

[...] *we had a strategic planning process, involving the management and senior leadership. They would meet to consider the institution’s strengths and weaknesses, how to analyze the internal environment; how to analyze the external environment; what threatened the achievement of our goals; what opportunities we had for improvement; what financial and material resources we had; and to set goals and indicators* [...]. (NBr-01)

*Nursing management has the autonomy to manage the process. I have complete autonomy within my group’s process to reach the nursing team, to decide where they go, and everything I need to do in terms of reassignment. There’s no need to consult another group. There is this aspect of autonomy.* (NBr-02)

*Today, I have the autonomy to make my own decisions. Today, I feel more confident in my decisions. I also went through a coaching process, which was very important for me, because leading people isn’t easy. Everyone thinks it’s wonderful, and you need to have a calling for it, which I do, but you also need help to stay grounded in the middle of a hurricane, because sometimes I felt like I was in the middle of a hurricane, constantly putting out fires.* (NBr-06)

In Spain, autonomy in decision-making is recognized, but there is a desire for greater independence, especially in managing work hours and direct supervision of teams. This indicates a search for greater flexibility and control over daily practices.

[...] *but then if I need more autonomy, for instance, in terms of managing working hours, the days I spend with my staff, I would like to be much more autonomous and not depend so much on the area’s supervision, for instance, and be able to give it to my staff.* (NSp-01)

In Portugal, the decision-making process is characterized by collaboration and shared responsibilities. Nurses mention the need to work collaboratively with supervisors on cross-disciplinary projects and the importance of building visibility and autonomy through respectful and humble interactions with other healthcare professionals.

*As for hierarchy* [...] *some guidance on cross-cutting projects involving various services falls under the control and supervision of the supervising nurse, and all of this must be shared and developed jointly. Therefore, if there are projects that cut across different services, we cannot develop them alone. We have to interact with the supervising nurse, monitor progress, and request their guidance.* (NPo-04)

*And therefore, this respect and this building of visibility and autonomy, I built it myself. I’ve been talking about this with my director, because of the contingency plan here, and I had to arrive late to a meeting because I had another meeting, and when I arrived, there were only doctors and administrators. Everyone asked me what I thought, and I said that one thing that worried me was the beds, and the clinical director said, “Well, there will be, there will be later, but I want the beds to be resolved before we go into the flu contingency plan, because I don’t have a flu contingency plan if I don’t have beds,” yes. And therefore, all this to say that I built my own visibility, my own autonomy, with humility, because I don’t* [...] *power is a strange thing.* (NPo-03)

These narratives demonstrate that, despite contextual differences, there is a common desire for greater autonomy and active participation in decision-making. However, there is a need to train nurses to understand autonomy less romantically, as if it were total freedom regardless of the decision-making process.

In relation to theory-driven NP, this is fundamental to critical, clinical, and managerial reasoning for professional autonomy. It acts as a tool for qualifying nurses’ care, enabling them to plan, implement, and assess care systematically and based on evidence. The experiences of nurses in Brazil, Spain, and Portugal illustrate how the NP influences their daily practice and autonomy.

In Brazil, nurses emphasize that autonomy is achieved through knowledge and professional respect. One Brazilian nurse reports that, in both private and public hospitals, respect was earned by demonstrating competence in their field. Another professional emphasizes that managing the care process involves prior organization and shared decision-making, allowing autonomy in daily life and deferring to management only in exceptional situations, demonstrating that autonomy is linked to the responsibilities already established within the organizational structure.

*I believe that nursing autonomy, we always say that we have to acquire it through our knowledge, by demonstrating our knowledge, and even when I was working in the private hospital, I had respect there, just as I have here, and I acquired that respect through my knowledge, by demonstrating it within my field. As a nurse, I had knowledge.* (NBr-02)

*My responsibility here is basically managing the patient care process, and this stage of the process involves prior organization of what can be offered to the patient* [...] *and from there, all decision-making is shared, so the team members have their voice, they can* [...] *their specific area of expertise, so that we can allocate resources in the best way possible, resources that we know are scarce in the healthcare field* [...] *on a daily basis, we have the autonomy to handle things independently; we only go to this team of more established managers when we go beyond what is our daily routine.* (NBr-05)

In Spain, nurses’ autonomy is characterized as complete in the healthcare field, although there are limitations in the financial sphere. However, this misunderstands professional autonomy. A Spanish nurse mentions having full autonomy in the nursing area, but not in the financial area. Another professional points out that, as long as they do not interfere in the medical area, they have autonomy, and decisions are made in consultation with service coordinators or directors.

*In the area that specifically concerns me, not in nursing. I have complete autonomy, but not as much in the financial area.* (NSp-09)

*From a nursing perspective, as long as it doesn’t involve the medical aspect, I obviously have autonomy. If it involves the medical aspect or the organization of the unit’s coordinating service or the service director, there is always an understanding. So far, I haven’t had any conflicts or disagreements regarding this. I know that this is not a very common reality, and I know that there is always one aspect or another that could be a source of disagreement in my current situation.* (NSp-10)

In Portugal, nurses’ autonomy is linked to the provision of care and the established hierarchy. A Portuguese nurse claims to have autonomy in decisions related to patient care. Another professional emphasizes that, although he reports directly to a nurse leader, he has autonomy in providing care and reports needs individually or in group meetings.

*I had autonomy in these decisions regarding the care process regarding patient care and everything related to them.* (NPo-07)

*In my job, I report directly to a nurse, who is the head nurse and manages the unit, and I have levels of autonomy in everything related to providing care. I report to her any needs that I or we identify, and in many cases, the reporting is done individually, and in others, in group meetings that we frequently hold in the department.* (NPo-08)

These narratives demonstrate that the NP provides nurses with a structure that facilitates autonomy in care practice, although factors such as hierarchy and financial resources can influence the degree of this autonomy. Understanding these dynamics is essential to strengthening professional practice and the quality of care provided.

Regarding the training processes related to the nursing management model, it is noteworthy that they play a crucial role in developing professionals’ autonomy and leadership. The experiences shared by nurses from Brazil, Spain, and Portugal provide an opportunity to better understand how training and practice influence their management skills.

In Brazil, a nurse reports a transition from a centralizing approach, linked to the scientific management model, to a more democratic and participatory approach that facilitates performance management for nursing workers. By delegating responsibilities and preparing administrative nurses to make decisions in her absence, she ensures management continuity and the development of her team’s leadership skills. This practice not only facilitates daily management but also prepares the team for future transitions and crises, enabling sustainable operations.

*I started to develop autonomy in my nurses, they tell me, because I used to be very centralized,* [...] *I like to have control of things, I like to know how things are going, but, at the same time, I like to give them autonomy so that everything isn’t centered only on me, because what I’ve learned is that I won’t be here forever. The time may come when I receive another offer for something else or when it’s no longer interesting for the institution to have me, in my position, and someone else will come, and I have to prepare myself. So, I have administrative nurses; that made all the difference, on weekends and holidays when I wasn’t at the hospital, so that was excellent because the girls can solve problems when I’m not at the hospital. They can also get a vision of what management is like, not just being a clinical nurse.* (NBr-06)

In Spain, a nurse emphasizes the need to improve the continuing training of healthcare professionals, especially those in management positions. He criticizes the stagnation of some supervisors who, after decades in the role, fail to update themselves as hospital settings and healthcare practices change. This observation highlights the importance of continuing education and adapting to the new realities of the sector.

*I think training needs to be improved for everything that was sent through the system, and the same applies to us here, where we are concerned about the student’s arrival and not about others. This has to be for all people in management positions. We cannot be supervisors for 25 years, living in the glory of the past, because 25 years ago, the hospital wasn’t like this, nor was healthcare in general.* (NSp-01)

Another Spanish nurse emphasizes autonomy in bed management and patient care organization. He mentions the freedom to decide on exams, care, and scheduling, as well as managing appointments and home visits. This autonomy is romanticized; however, we understand that autonomy regulated by institutional protocols that promote more effective workflows can enable more precise adaptation to patient needs and more efficient management of available resources.

*For instance, to manage the beds, where one hospitalization goes, where another goes, we can do this, when, for instance, we decide certain exams or certain care, what time they are done, or what time they are not, tell the doctor, well, is it better this way or not, to organize my work I am in the hospital.* [...] *it is totally autonomous because you have your consultation, you manage, you have your schedule,* [...] *when you go to see a house, you manage everything there.* (NSp-08)

In Portugal, nursing student training is enriched by mentoring programs that aim to develop clinical, theoretical, and pedagogical skills. A Portuguese nurse emphasizes the importance of clinical supervisors being qualified not only in technical knowledge but also in pedagogy, policy, administration, and communication, to better guide students and developing professionals. This holistic approach ensures that future nurses are well prepared for the challenges of professional practice.

*The role of tutors or mentors in student training processes, whether in the initial development pathway or in the advanced pathways. To achieve this, it’s necessary not only to understand the clinical perspective, from a theoretical nursing perspective, but also to understand pedagogy, didactics, and communication. Our goal with this clinical supervisor training was essentially to help these nurses develop skills in this dimension I mentioned, to become better communicators, better equipped to communicate and teach students and other professionals in the advanced development course.* (NPo-02)

Another Portuguese nurse mentions having a training professional within the team, who plans and develops qualifications based on identified needs. This practice promotes continuous team development and ensures that necessary skills are constantly updated.

*We have a nurse on our team who is responsible for team training. We plan and develop this training ourselves. Obviously, we provide the necessary knowledge and ask for direct help in specific situations, but we have the autonomy to identify our team’s needs and promote that development.* (NPo-04)

These narratives highlight the importance of continuous and adaptive training processes in strengthening nursing management. Promoting autonomy, constant updating, and building leadership and communication skills are essential for effective practice aligned with contemporary healthcare demands.

## DISCUSSION

It is noted that the hospitals in the three countries studied have non-uniform characteristics in the investigated settings, which is explained by differences in the management model adopted and, consequently, in the exercise of supervision, as well as by economic, political and social factors that interfere in care management and in professional autonomy construction/strengthening.

It is important to note that nurses’ professional autonomy in hospital settings is a complex issue that has been changing over time, according to the historical and social context. It is observed that, due to the influence of the prevailing biomedical model in this environment, there is a fragility in professional autonomy associated with professional identity and the idea of subservience throughout this historical context. The definition of professional autonomy is considered a powerful ideology, which involves the idea of regulated authority to make decisions and freedom to act within one’s professional practice, associating experience and knowledge with this decision-making. Furthermore, it interfaces with work experience through participation in decision-making and the capacity for change in work processes^(15)^.

In this regard, it is also important to consider the concept of nursing autonomy, which allows nurses to practice at the highest level of training and scientific knowledge to provide quality healthcare, being a significant factor in job satisfaction for nurses^(7)^. Beliefs based on medical practice confuse this concept in nurses’ practice and their object of care. The concept of autonomy in the field of education brings to the discussion the respect for human rights and ethics in the context of the teaching-learning process and in educational relationships^(8)^. It is from this perspective that this assumption resembles the concept of social supervision, which understands the educational dimension of supervision in the pursuit of workers’ development as co-responsible in healthcare production^(10)^.

Supporting this idea, it is noteworthy that nurses have as their object of work patients’ holistic care, in defense of life, being the scope of their professional practice. Furthermore, in countries like Portugal and Spain, the management model adopted shows strong levels of hierarchy in the organizational power structure that limit the contribution of supervision to the construction/strengthening of autonomy. This became clear during the analysis of speech fragments, which demonstrate the operational levels and the power established in the exercise of supervision.

A study discussed autonomy and meaningful work, concluding that both are positively related to job satisfaction levels for nurses, as are working conditions, recognition, and professional appreciation^(16)^. The understanding of meaningful work is related to the meaning of work within individuals’ worldview. This study shows that the development of professional autonomy demands the alignment of supervisory hierarchy levels within the power structure from the perspective of participatory management. Supervision is recognized as a management tool that fosters the development of the nursing team’s potential and as a strategy aimed at meeting the training needs for clinical practice, in addition to contributing to professional autonomy construction/strengthening.

Nurses’ decision-making process differed in the three countries. In Brazil, it was aligned with the adopted management model and supported by the management tools used, including strategic planning and supervision. The presence of traditional supervision was also observed, as well as encouragement of participatory management, encouraging shared responsibility among the actors involved, from a co-management perspective, characteristics of social supervision. The study analyzes the development of autonomy through nurse leadership and conditions linked to participatory management, especially regarding the creation of conditions for maintaining a healthy workforce^(17)^. Thus, job satisfaction is considered a predictive element for the retention of this workforce in the healthcare organization and, indeed, in the profession itself.

However, the results demonstrated the power of a participatory decision-making process focused on sharing knowledge and power, recognizing interand multidisciplinarity as more effective strategies in the bounded rationality decision-making model, where satisfactory decisions are not necessarily the best ones.

Autonomy development/strengthening through leadership is addressed in a study that highlights the emergence of a new type of leader, with characteristics focused on seeking a balance between autonomy and responsibility, emphasizing teamwork and the improvement of healthcare outcomes^(18)^. Although this same concern has not been observed in Portugal and Spain, it is noted in these countries that the decision-making process is controlled and, consequently, autonomy is more regulated. There is a perceived need to direct efforts towards strengthening clinical supervision from the initial training of nurses to underpin the provision of quality and safe care for patients^(19)^. In this context, supervision is considered a management tool for adjustment between the operational area and strategic management, in order to facilitate the achievement of organizational objectives.

In turn, the production of care from the perspective of theory-driven NP is a potentiality related to the contribution of supervision to the construction/strengthening of nurses’ autonomy in hospital settings in Ibero-American countries, which signals and recognizes the NP as a method in care production, requiring from nurses to possess scientific knowledge, clinical judgment, critical thinking, commitment to others, and responsibility in decision-making. Nursing professionals’ autonomy must be consolidated through the use of the systematization of nursing care. This includes clinical judgment and critical thinking, which underpin nurses’ decision-making, as well as the concept of responsibility, without restrictions in professional collaboration with other health professionals within the scope of their professional practice^(7)^.

From this same perspective, autonomy is considered to present constitutive elements for understanding teaching practice as a social dimension of human formation^(8)^, being an important postulate for reflecting on actions that lead to fragility in the provision of healthcare services. This thinking is related to the theme of nursing supervision defended in this research, understanding supervision as a social practice that is involved in educational processes^(10)^. A study found that the recognition of nurses’ autonomy is related to the Nursing Care Systematization implementation^(20)^. It is considered a path to building/developing/strengthening autonomy for the profession, as it represents a way of organizing nursing care recognized by nurses, which allows for the management of care with patients, by requiring scientific knowledge, professional responsibility, and commitment to professional practice.

Furthermore, training processes related to the nursing management model are a potential aspect of supervision that contributes to the construction/strengthening of nurses’ autonomy in hospital settings in Ibero-American countries, which present differences in how they are conducted in the investigated settings. From this perspective, the importance of promoting educational practices and knowledge management in healthcare services is emphasized, as well as the development of autonomy among team members and the encouragement of organizational growth, and the development of managerial competencies for the exercise of nursing supervision^(21)^.

Based on the analysis of the selected excerpts from the interviews, especially those from Portugal and Spain, it is evident that training processes are driven by errors or non-conformities that occur in the production units, characteristic of traditional supervision. This finding reiterates the need to provide better professional training in the monitoring of people, care, services, and health systems in favor of supervision focused on organizational and personal development, paying more attention to the 2030 agenda Sustainable Development Goals. However, the training process is discussed with the following reflection: “*It is in this sense that I insist that training is much more than purely teaching*”^(8)^.

Analyzing this perspective, one observes a concern within the services to offer courses, in-service training, and continuing education previously tailored to the service’s needs or real-world problems and cases. However, there is a need to rekindle the curiosity of these workers, so that the pursuit of knowledge is constant. It is from this perspective that social supervision defends the educational dimension, with workers as leading actors of this process.

The critical-reflective dimension, as well as the socio-political dimension, is often neglected in the nursing team’s work process, and this has serious impacts on the production, meaning, quality, and humanization of respectful care. The political dimension is essential for valuing and recognizing practices that promote quality of life, projects for happiness, and a harmonious and healthy society.

It is worth noting that, in Brazil, some aspects related to the formative process aimed at technical preparation and the potential of supervision have been identified that contribute to and strengthen the construction of autonomy in relation to production unit management and nurses’ work process in hospital settings. A study concluded that, in the last decade, nurses’ autonomy within the health work process has been strengthening. This allows them to act in advanced practices, aiming not only to increase access to care for users, but also to mitigate the costs of healthcare services^(22)^.

### Study limitations

This study presents some limitations that should be considered when interpreting the results. Firstly, the research was conducted in hospitals in only three Ibero-American countries - Brazil, Spain, and Portugal - which may not reflect the reality of other nations or regions with different socioeconomic and cultural contexts. Furthermore, the selection of participants was limited to nurses working in specific hospital settings, which may not represent the diversity of experiences and practices existing in other healthcare contexts.

### Contributions to nursing, health, or public policy

This study offers significant contributions to the field of nursing, especially with regard to understanding the strengths and weaknesses of supervision in building and strengthening the autonomy of nurses in hospital settings. The research provides an opportunity to understand and reflect on nursing management practices and models in different Ibero-American countries, which can support the implementation of more effective supervision strategies aligned with nursing professionals’ needs. Furthermore, by highlighting the importance of training processes and the NP, the study reinforces the need for continuous investment in nurses’ continuing education, aiming to improve the quality of care provided and promote professional autonomy. Finally, by identifying limitations and weaknesses in current supervision models, the research paves the way for future investigations that can delve deeper into these topics, contributing to the development of more participatory and empowering supervision practices in nursing.

## FINAL CONSIDERATIONS

In conclusion, the study achieved its proposed objective, which was to analyze nursing supervision contributions and intersections, identifying a transformative social practice with implications for systems of care for individuals, families, and communities, in the pursuit of developing the worker as an element of shared vision and responsibility in care production. Thus, this study provided a better understanding of the development of nursing supervision in building/strengthening professional autonomy in hospital settings in Ibero-American countries, with specific nuances in the field of limitations and potentialities, understanding that the management model adopted is influenced by cultural, social, economic, and political factors.

## Data Availability

The research data are available only upon request.
