# Supplementary material for: Nurses’ supervision and autonomy in hospitals: an Ibero-American multicenter study
Source: Rev Bras Enferm. 2026 Jul 27;79:e20250278. doi: 10.1590/0034-7167-2025-0278 (PMC13411321; doi:10.1590/0034-7167-2025-0278)
Supplement: Translation

# Translation: Supervisão e autonomia do enfermeiro no contexto hospitalar: estudo multicêntrico ibero-americano

**DOI:** 10.1590/0034-7167-2025-0278pt

**Authors:** Rebecca Maria Oliveira de Góis, Gilberto Tadeu Reis da Silva, Maria Lúcia Silva Servo, Thadeu Borges Souza Santos, Edenise Maria Santos da Silva Batalha, Monica Motta Lino, Silvana Lima Vieira, Deybson Borba de Almeida

**Affiliations:** I Universidade Federal da Paraíba. João Pessoa, Paraíba, Brasil; II Universidade Federal da Bahia. Salvador, Bahia, Brasil; III Universidade Estadual de Feira de Santana. Feira de Santana, Bahia, Brasil; IV Universidade Estadual da Bahia. Salvador, Bahia, Brasil; V Universidade Federal de Santa Catarina. Florianópolis, Santa Catarina, Brasil

**Keywords:** Enfermagem, Supervisão de Enfermagem, Autonomia Profissional, Ambiente Hospitalar, Estudo Multicêntrico.

## Abstract

**Objetivos:**

analisar os contributos e atravessamentos da supervisão do enfermeiro na construção e implementação da autonomia profissional regulada de equipes de enfermagem em ambientes hospitalares de países ibero-americanos.

**Métodos:**

pesquisa qualitativa, oriunda de estudo multicêntrico, realizado em hospitais universitários de Portugal, Espanha e Brasil. Foram conduzidas entrevistas semiestruturadas com enfermeiros assistenciais e gestores de internação. A análise de conteúdo foi utilizada para interpretar os dados coletados.

**Resultados:**

a supervisão apresentou variações nos três países, influenciada por contextos organizacionais e culturais. A supervisão e sua potencialidade para o desenvolvimento da autonomia foram influenciadas pela hierarquia institucional, estrutura dos processos decisórios e processos formativos. Uma abordagem de supervisão baseada no apoio e desenvolvimento profissional favorece a autonomia dos enfermeiros, enquanto práticas centradas no controle podem limitá-la.

**Considerações Finais:**

a ênfase em modelos de supervisão que promovem o desenvolvimento e a participação dos enfermeiros contribui para práticas assistenciais mais autônomas e eficazes.

## INTRODUÇÃO

A supervisão está intrinsecamente atrelada ao processo de trabalho do enfermeiro. Nota-se que a prática supervisiva no ambiente hospitalar possui *interface* com o modelo de gestão adotado para alcance dos objetivos e metas estratégicas, bem como alavancar os padrões de qualidade e de acreditação nos serviços de saúde, além de implicar a *performance* dos profissionais^(1)^. Nesse contexto, a supervisão é de fundamental importância na tomada de decisão autônoma dos enfermeiros e na oferta de cuidados seguros. Além de estimular a integração e motivação das equipes, contribui para produção de cuidados mais eficientes e humanizados em ambientes hospitalares^(2,3)^.

O processo de supervisão, juntamente com o planejamento e a avaliação, constitui uma tecnologia essencial para a gestão dos serviços de saúde, alinhando a missão, a visão, os valores e os objetivos da organização com as atividades diárias dos trabalhadores no contexto hospitalar. Técnicas de monitoramento e acompanhamento das equipes, como *feedback* construtivo, reuniões, avaliação de desempenho do tipo 360 graus, análise de casos e supervisão a distância, construídas com técnicas grupais e individuais, têm sido amplamente utilizadas para otimizar a supervisão e promover melhorias no cuidado em saúde. Salienta-se que os principais instrumentos utilizados no processo de supervisão do enfermeiro são prontuários, plano de cuidados, processo de enfermagem (PE) guiado por uma teoria, plano de supervisão, cronograma de trabalho, estabelecimento de indicadores de monitoramento e avaliação, o método Deming para melhoria da qualidade, além dos fluxos, linhas de cuidado, e manuais/regimentos do serviço de saúde/enfermagem com as instruções de normas e rotinas^(4,5)^.

Nota-se que a atuação do profissional que exerce essa função exige a mobilização de um conjunto de competências e habilidades que envolve conhecimentos técnicos, administrativos e políticos específicos, e a habilidade de implementar a gestão da *performance* da organização e das pessoas da equipe de enfermagem, contribuindo, assim, para construção/fortalecimento da autonomia profissional^(6,7)^.

Nesse quesito, é importante afirmar que a autonomia é uma característica sempre regulada no trabalho em equipe, pois a autonomia de todo e qualquer profissional é relativa ao arcabouço político, jurídico e institucional, nomeadamente os protocolos clínicos, as diretrizes terapêuticas, as leis sanitárias, as regulamentações de conselhos profissionais e as políticas do Sistema Único de Saúde.

Contudo, a supervisão é fundamental para o fortalecimento da autonomia profissional, mesmo que regulada, compreendida como a possibilidade do enfermeiro de tomar decisões independentes dentro de um sistema tácito instituído, exercendo suas responsabilidades de forma efetiva. Estudos apontam que práticas supervisivas estruturadas implicam a confiança, a habilidade de resolução de problemas e a competência técnica dos profissionais, oportunizando não apenas a melhoria da qualidade do cuidado, mas também o desenvolvimento da sua *performance* como líder e ordenador do cuidado^(8,9)^. Além disso, abordagens interativas, como o uso de metodologias ativas e tecnologias avançadas, têm mostrado resultados promissores na formação de enfermeiros para contextos complexos^(10)^.

A construção e implementação da autonomia do enfermeiro também estão relacionadas ao fortalecimento de saberes e competências ao longo do processo formativo. Reflexões sobre sua identidade e seu processo de trabalho, bem como o significado de autonomia, devem ser incentivadas desde a graduação, promovendo uma prática profissional mais efetiva e voltada à tomada de decisão compartilhada^(9-11)^. Sob essa ótica, a supervisão é vista como um dispositivo pedagógico que não apenas orienta tecnicamente, mas também contribui para a gestão da *performance* pessoal e profissional dos membros da equipe^(12)^. Adicionalmente, estudos recentes reforçam que a supervisão, quando realizada de forma estruturada, desempenha papel crucial nas políticas de gestão de pessoas e no desenvolvimento de ambientes de trabalho resilientes e respeitosos^(13)^.

Com base no exposto, o presente estudo busca analisar os contributos e atravessamentos da supervisão do enfermeiro na construção e implementação da autonomia profissional regulada em ambientes hospitalares de países ibero-americanos. Ao abordar diferentes contextos históricos, culturais, sociais e políticos da enfermagem, este trabalho espera refletir sobre potencialidades, lacunas e dificuldades neste processo de supervisão do enfermeiro em prol de práticas de cuidado que dialoguem com modelos mais equânimes, eficientes e eficazes.

### Relevância do estudo

Este artigo é produto de tese^(14)^ intitulada “Processo de supervisão do enfermeiro: contributos para o desenvolvimento da autonomia no ambiente hospitalar”, que está depositada no repositório institucional através do *link:* https://repositorio.ufba.br/handle/ri/37325.

## OBJETIVOS

Analisar os contributos e atravessamentos da supervisão do enfermeiro na construção e implementação da autonomia profissional regulada de equipes de enfermagem em ambientes hospitalares de países ibero-americanos.

## MÉTODOS

### Aspectos éticos

Esta pesquisa foi aprovada pelo Comitê de Ética em Pesquisa. Está vinculada ao projeto matriz “Modelos de Gestão Hospitalar em Enfermagem: memórias de enfermeiros”. Todos os participantes assinaram o Termo de Consentimento Livre e Esclarecido, garantindo a sua participação de forma voluntária, autônoma, consciente, livre e esclarecida. Os princípios éticos da e da Lei n^o^ 1.4874/2024 e normas éticas internacionais foram respeitados. Para preservar a identidade, foram adotados pseudônimos aos participantes com a letra “E” de enfermeiro, seguida de “Br”, para Brasil, “Es”, para Espanha, e “Po”, para Portugal, além de um número.

### Tipo de estudo

Estudo multicêntrico de abordagem qualitativa, descritivo e exploratório, realizado em três países ibero-americanos (Brasil, Portugal e Espanha). As diretrizes do guia *COnsolidated criteria for REporting Qualitative research* foram seguidas reforçando o rigor metodológico para pesquisas qualitativas.

### Procedimentos metodológicos

A partir do roteiro semiestruturado com dados de caracterização, como tempo de formação e qualificação profissional, foram elaboradas as perguntas norteadoras. Nesse sentido, vale ressaltar que a ênfase deste estudo residiu em aprofundar a discussão no intuito de analisar a contribuição da supervisão do enfermeiro para construção/fortalecimento da autonomia profissional no ambiente hospitalar em países ibero-americanos.

### Cenário do estudo

O estudo foi desenvolvido em hospitais universitários localizados na Bahia (Brasil), em Coimbra (Portugal) e Toledo (Espanha). Essas instituições foram escolhidas por representarem cenários de ensino, pesquisa e assistência, bem como por refletirem diferentes modelos de gestão em enfermagem e saúde.

### Fonte de dados

Participaram da pesquisa 30 enfermeiros, sendo nove brasileiros, oito espanhóis e 13 portugueses. Foram incluídos enfermeiros que atuam ou atuaram como gestores de topo, intermediários ou gerentes de unidade/serviço com, pelo menos, seis meses de experiência na função. Foram excluídos enfermeiros em férias, licença-maternidade ou afastamento por doença no período de coleta de dados, bem como aqueles que não retornaram o contato após três tentativas de agendamento ou que recusaram participação.

### Coleta e organização dos dados

A coleta de dados foi realizada por meio de entrevistas semiestruturadas, conduzidas entre setembro de 2020 e fevereiro de 2021, em salas reservadas nos hospitais. As entrevistas foram baseadas em roteiro previamente validado e composto por cinco questões norteadoras, abordando aspectos sociodemográficos, modelos de gestão organizacional e práticas de supervisão. As perguntas exploraram tópicos como processo decisório, avaliação de desempenho, planejamento de ações de gestão e processos educativos. As entrevistas, com duração média de 60 a 120 minutos, foram gravadas e transcritas. A seleção dos participantes utilizou a técnica *snowball sampling*, em que os enfermeiros indicavam outros potenciais participantes com base em redes de referência. Após a transcrição para validação das entrevistas com as participantes, procedeu-se à saturação de dados, constatando-se a necessidade de encerramento da colheita de material por não aparecer aspectos novos no rol das respostas; por isso, a coleta foi encerrada.

### Análise de dados

Ocorreu mediante análise temática do conteúdo, e contemplou as seguintes etapas: (i) exploração inicial: compreensão do contexto sócio-histórico dos grupos sociais analisados; (ii) ordenação dos dados: sistematização das informações coletadas e organização das falas em núcleos de sentido; e (iii) classificação dos dados: construção de inferências e interpretações com base nos fundamentos teóricos do estudo. Os *softwares* NVivo^®^ 11 e *webQDA* ^®^ foram utilizados para organizar os dados e facilitar a análise qualitativa. A análise temática identificou uma categoria principal: “Potencialidades relacionadas à contribuição da supervisão para desenvolvimento/fortalecimento da autonomia do enfermeiro no ambiente hospitalar”.

## RESULTADOS

Destaca-se no Quadro 1 a caracterização dos participantes segundo idade, sexo, estado civil, cor da pele e religião. Constatou-se uma maior frequência do sexo feminino, estado civil casado e religião católica nos três países. Quanto à idade, em Portugal, a maioria tinha de 51 a 60 anos, enquanto no Brasil e Espanha, de 30 a 40 anos.

**Quadro 1:** Caracterização de informações sociodemográficas dos sujeitos entrevistados no Brasil, Portugal e Espanha (2020) no Hospital Universitário Professor Edgard Santos (Salvador, Bahia, Brasil), hospitais da Universidade de Coimbra (Coimbra, Portugal) e Complexo Hospitalar Universitário de Toledo (Toledo, Espanha)

INFORMAÇÕES SOCIODEMOGRÁFICAS		PORTUGAL	ESPANHA	BRASIL
**Idade**	De 30 a 40 anos	2	3	4
	De 41 a 50 anos	1	0	2
	De 51 a 60 anos	7	2	2
	A partir dos 61 anos	3	1	1
	Não informado	0	2	0
**Sexo**	Feminino	9	7	8
	Masculino	4	1	1
**Estado civil**	Solteira(o)	2	1	2
	Casada(o)	9	4	6
	Divorciada(o)	1	0	1
	Viúva(o)	1	0	0
	Não informado	0	3	0
**Cor da pele**	Branca	7	0	2
	Negra	0	0	2
	Parda	0	0	5
	Caucasiana	1	0	0
	Não informado	5	8	0
**Religião**	Católica	9	3	5
	Cristã	0	0	1
	Evangélica	0	0	1
	Espírita	0	0	1
	Não possui religião	0	0	1
	Protestante	2	0	0
	Ateu	1	0	0
	Não informado	1	5	0

Além disso, evidencia-se a caracterização dos participantes segundo escolaridade e tempo de formação no Brasil, Portugal e Espanha. Em relação à escolaridade nos três cenários investigados, nota-se que há discrepância entre os países, como evidenciado no Quadro 2.

**Quadro 2:** Caracterização de informações acadêmicas dos sujeitos entrevistados no Brasil, Portugal e Espanha (2020) no Hospital Universitário Professor Edgard Santos (Salvador, Bahia, Brasil), hospitais da Universidade de Coimbra (Coimbra, Portugal) e Complexo Hospitalar Universitário de Toledo (Toledo, Espanha)

INFORMAÇÕES ACADÊMICAS		PORTUGAL	ESPANHA	BRASIL
**Escolaridade**	Superior com pós-graduação/especialização	5	3	5
	Licenciatura em enfermagem	1	0	0
	Superior com mestrado	7	2	1
	Superior com doutorado	0	1	2
	Pós-doutorado	0	0	1
	Não informado	0	2	0
**Tempo de formação**	5 a 10 anos	0	0	1
	11 a 20 anos	2	3	4
	21 a 30 anos	2	2	2
	31 a 37 anos	6	3	2
	38 a 40 anos	1	0	0
	41 a 50 anos	2	0	0
**Instituição de formação em nível superior**	Universidade Federal da Bahia (pública)	0	0	5
	Universidade Federal do Rio Grande do Sul (pública)	0	0	1
	Universidade Católica de Salvador (privada)	0	0	2
	Universidade Jorge Amado (privada)	0	0	1
	Escola Superior de Enfermagem Dr. Ângelo da Fonseca	6	0	0
	Escola Superior de Enfermagem de Coimbra	1	0	0
	Escola Superior de Enfermagem de Sá Barreto	2	0	0
	Escola Superior de Enfermagem de Bissaya Barreto	4	0	0
	Universidade a distância	0	1	0
	*Universidad de Castilla-La Mancha*	0	1	0
	*Universidade Complutense de Madrid*	0	1	0
	*Universidad Rovira i Virgili*	0	1	0
	*Universidad Autónoma de Madrid*	0	1	0
	Não informado	0	3	0

Além disso, evidencia-se no Quadro 2 a caracterização dos participantes segundo escolaridade, tempo de formação e instituição formadora. Em relação à escolaridade nos três cenários investigados, nota-se que há discrepância entre os países, sendo que, em Portugal, a maioria possuía título de mestre, e na Espanha e no Brasil, título de especialista. Relativo ao tempo de formação, em Portugal destaca-se um tempo superior a 30 anos, diferindo dos participantes da Espanha e do Brasil. Destaca-se a Figura 1, que sintetiza o processo de coleta de dados até a elaboração das categorias do estudo.

**Figura 1:** Síntese da categoria sobre a contribuição da supervisão do enfermeiro para construção/fortalecimento da autonomia profissional no ambiente hospitalar em países ibero-americanos (Salvador, Bahia, Brasil; Coimbra, Portugal; Toledo, Espanha)

### Potencialidades relacionadas à contribuição da supervisão para desenvolvimento/fortalecimento da autonomia do enfermeiro no ambiente hospitalar

A autonomia do enfermeiro no ambiente hospitalar é influenciada por diversos fatores, como o modelo de gestão e atenção em saúde, representado muitas vezes na estrutura hierárquica da supervisão dentro da organização. As falas dos participantes de Portugal, Espanha e Brasil refletem diferentes perspectivas sobre como essa hierarquia afeta sua autonomia profissional.

No contexto brasileiro, observa-se uma supervisão que apoia as lideranças de unidade por meio de figuras como o “enfermeiro de referência”, proporcionando suporte direto às equipes. Um dos enfermeiros brasileiros destaca a autonomia concedida pelas gestoras intermediárias, que permite ao profissional gerir suas demandas e equipe, com a gestão oferecendo apoio e mantendo uma comunicação aberta.

[...] *essa autonomia dentro de cada unidade, esse apoio da divisão, a gente tem uma pessoa que responde por essa supervisão, então é como se fosse hoje a pessoa que responde, né, por apoiar, essas chefias de unidade, ele chama, na verdade, hoje de enfermeiro de referência.* (EBr-03)

*Eu estou vivendo o que eu sempre vivi, o que eu acho que é o certo* [...] *a chefia que te dá total autonomia, você a gestora, você que vai saber como é que vai agir, que você conhece suas demandas,* [...] *sua equipe,* [...] *você vai fazer o melhor, eu só quero o seu retorno.* [...] *uma gestão que apoia muito a gente, que ouve muito a gente. Então, hoje eu chego lá na divisão a qualquer momento. Eu sou super bem-vinda, a porta está sempre aberta, não existe “ah, não venha agora porque eu estou super ocupada”. Eu me sinto muito mais acolhida, e hoje eu tenho muito mais autonomia, então isso refletiu muito mais diretamente no meu trabalho, na minha equipe.* (EBr-06)

Na Espanha, a autonomia é percebida no nível profissional, permitindo que os enfermeiros tomem decisões dentro de parâmetros éticos e práticos estabelecidos. Um enfermeiro espanhol menciona que, embora exista autonomia, ela está condicionada ao cumprimento de normas éticas e práticas institucionais.

*Sim, há autonomia, mas a nível profissional, diríamos, pelo seu trabalho direto, se houver algum outro, desde que não faça nada que não seja ético ou algo errado, é práxis, mas se há uma certa autonomia, há uma certa autonomia.* (EEs-05)

Em Portugal, a supervisão é monitorada no nível operacional, com enfermeiros-chefes e diretores de serviço responsáveis por indicadores específicos. Os profissionais portugueses enfatizam a importância de resolver questões internamente, recorrendo à hierarquia apenas quando necessário, o que demonstra uma abordagem colaborativa e focada na resolução de problemas dentro das próprias equipes.

*Existe uma monitorização ao nível operacional, ou seja, que é o enfermeiro-chefe, o diretor de serviço que tem que responder pelos indicadores que contratualizaram para o seu serviço.* (EPo-02)

*Nós só transpomos para a hierarquia tudo aquilo que de fato não conseguimos resolver internamente ou pensamos que podemos ter um contributo importante para uma decisão mais sustentada.* (EPo-04)

*Depende do problema que for, normalmente não coloco questões ao supervisor ao menos que seja alguma coisa que envolva a instituição, uma reclamação grave, um incidente grave, obviamente o conhecimento. Agora, questões pontuais do dia a dia, só se eu conseguir resolver.* (EPo-10)

Essas diferentes perspectivas evidenciam como a estrutura hierárquica e a cultura organizacional em cada país influenciam a autonomia dos enfermeiros no ambiente hospitalar. Compreender essas nuances é fundamental para aprimorar práticas de supervisão que fortaleçam a autonomia profissional.

Quanto ao processo decisório dos enfermeiros no ambiente hospitalar, é moldado por diversos fatores, incluindo estrutura organizacional, autonomia profissional e práticas de liderança. As experiências compartilhadas por enfermeiros do Brasil, Espanha e Portugal oferecem uma visão abrangente de como esses elementos influenciam suas decisões diárias, porém é possível constatar que esse processo é contingencial e, por vezes, assistemático e monocrático.

No Brasil, os enfermeiros destacam a importância do planejamento estratégico institucional, que envolve identificação de pontos de potência e limitações, análise de ambientes internos e externos, e definição de metas e indicadores. Além disso, enfatizam a autonomia na gestão de suas equipes e a necessidade de desenvolvimento pessoal, como a participação em processos de *coaching*, para fortalecer a tomada de decisões e a liderança eficaz.

[...] *nós tínhamos um planejamento estratégico, a direção, a alta administração. Ela se reunia pra poder pensar quais eram os pontos fortes institucionais, quais eram as fragilidades, como é olhar para o ambiente interno; olhar para o ambiente externo; olhar o que é que ameaçava o alcance das nossas metas; olhar quais eram as possibilidades que nós tínhamos de melhoria; olhar os recursos financeiros, os recursos materiais; traçar metas, indicadores* [...]. (EBr-01)

*A gestão de enfermagem, ela tem autonomia de ter o processo. Eu tenho total autonomia dentro do processo do meu grupo de chegar ao grupo de enfermagem, de decidir para onde eles vão, todo o processo do que eu tenho que fazer de remanejar. Não tem essa questão de ter que procurar saber de outro grupo. Há essa questão da autonomia.* (EBr-02)

*Hoje, eu tenho autonomia de tomar as minhas decisões. Hoje, eu me sinto mais firme nas minhas decisões. Eu passei também por um processo de coaching que, para mim, foi muito importante, porque a gente liderar pessoas não é uma coisa fácil, né, todo mundo acha que é lindo, e você precisa ter vocação, eu gosto, mas você precisa também de uma ajuda para você conseguir se segurar no meio de um furacão, porque, às vezes, eu sentia que eu estava no meio de um furacão, que eu ficava apagando incêndio.* (EBr-06)

Na Espanha, a autonomia no processo decisório é reconhecida, mas há um desejo de maior independência, especialmente na gestão de jornadas de trabalho e na supervisão direta das equipes. Isso indica uma busca por maior flexibilidade e controle sobre as práticas diárias.

[...] *mas aí, se eu precisar de mais autonomia, por exemplo, ao nível da gestão das jornadas de trabalho, dos dias que vou passar com o meu pessoal, gostaria de ser muito mais autônomo e não depender tanto no exemplo da fiscalização da área e poder dar para o meu pessoal.* (EEs-01)

Em Portugal, o processo decisório é caracterizado pela colaboração e compartilhamento de responsabilidades. Os enfermeiros mencionam a necessidade de trabalhar em conjunto com supervisores em projetos transversais e a importância de construir visibilidade e autonomia por meio de interações respeitosas e humildes com outros profissionais de saúde.

*Enquanto hierarquia* [...] *algumas orientação de projetos transversais de diversos serviços sobre controle e supervisão do enfermeiro supervisor, e tudo isso tem que ser partilhado e desenvolvido em conjunto, então, se há projetos transversais aos serviços, nós não podemos desenvolver sozinho. Temos que interagir com o enfermeiro-supervisor, acompanhar e solicitar a sua orientação.* (EPo-04)

*E, portanto, esse respeito e esta construção de visibilidade e de autonomia, eu fui construindo-a. Tenho falado isso com minha diretora, por causa do plano de contingência daqui, e eu tinha que chegar em uma reunião atrasada porque eu tinha outra reunião, e quando eu cheguei, estavam só médicos, administradores. Todo mundo me perguntou o que eu achava, e eu disse uma coisa que me preocupa eram as camas, e o diretor clínico disse: “Pois estará, pois depois tem, mas eu quero que as camas sejam resolvidas antes de entrarmos em contingência da gripe, porque eu não tenho plano de contingência da gripe se eu não tiver camas?”, sim. E, portanto, isso tudo para dizer que eu construí a minha própria visibilidade, da minha própria autonomia, da humildade, porque eu não* [...] *o poder é uma coisa estranha.* (EPo-03)

Essas narrativas evidenciam que, embora existam diferenças contextuais, há uma busca comum por maior autonomia e participação ativa no processo decisório. Contudo, identifica-se a necessidade de qualificar os enfermeiros diante da necessidade da compreensão menos romantizada da autonomia, como se fosse uma liberdade total a despeito do processo de tomada de decisão.

No tocante ao PE guiado por uma teoria, esta é fundamental ao raciocínio crítico, clínico e gestor para uma autonomia profissional que atue como dispositivo de qualificação do cuidado dos enfermeiros, permitindo-lhes planejar, executar e avaliar os cuidados de forma sistemática e baseada em evidências. As experiências de enfermeiros do Brasil, Espanha e Portugal ilustram como o PE influencia sua prática diária e autonomia.

No Brasil, os enfermeiros destacam que a autonomia é conquistada por meio do conhecimento e respeito profissional. Um enfermeiro brasileiro relata que, tanto em hospitais privados quanto em públicos, o respeito foi adquirido, demonstrando competência em sua área. Outro profissional enfatiza que a gestão do processo assistencial envolve organização prévia e decisões compartilhadas, permitindo autonomia no cotidiano e recorrendo às chefias apenas em situações excepcionais, demonstrando que a autonomia está relacionada às atribuições já instituídas dentro da estrutura organizacional.

*Acho que a autonomia de enfermagem, a gente sempre diz que tem que adquirir com nosso conhecimento, mostrando nosso conhecimento, que até quando estava trabalhando eu tinha respeito no hospital privado como tenho aqui, e esse respeito eu adquiri com meu conhecimento, de mostra dentro da minha área. Como enfermeira, eu tinha conhecimento.* (EBr-02)

*A gestão que me compete aqui é uma gestão basicamente do processo assistencial que a gente tem para o paciente, e aí essa etapa do processo ela envolve uma organização prévia do que pode ser oferecido para paciente* [...] *e a partir daí, as tomadas de decisão são todas compartilhadas, então os membros da equipe têm a sua voz, eles conseguem* [...] *a sua área de atuação específica, para a gente poder alocar, da melhor maneira, recursos que a gente sabe que na área de saúde são poucos* [...] *o dia a dia a gente tem autonomia de tocar sozinho, só vai pra essa equipe de chefias, né, mais estabelecidas, quando a gente sai do que é o nosso dia a dia*. (EBr-05)

Na Espanha, a autonomia dos enfermeiros é caracterizada como plena no âmbito assistencial, embora haja limitações na esfera financeira. No entanto, isso é uma incompreensão da autonomia profissional. Um enfermeiro espanhol menciona ter total autonomia na área da enfermagem, mas não na financeira. Outro profissional aponta que, desde que não interfira na área médica, possui autonomia, e as decisões são tomadas em entendimento com coordenadores ou diretores de serviço.

*Na área que me diz especificamente respeito, na área da enfermagem não. Tenho autonomia total, só não tenho tanto na área financeira.* (EEs-09)

*Do ponto de vista da enfermagem, desde que não implique a parte médica, tenho obviamente autonomia. Se implicar à parte médica ou organização do serviço coordenador da unidade ou ao diretor do serviço, sempre há um entendimento. Até o momento, não tive nenhum conflito, nenhum descontentamento relativo a isto. Sei que não é uma realidade muito comum, sei que há sempre um aspecto ou outro que poderá ser motivo de discórdia na minha realidade atual.* (EEs-10)

Em Portugal, a autonomia dos enfermeiros está relacionada à prestação de cuidados e à hierarquia estabelecida. Um enfermeiro português afirma ter autonomia nas decisões relacionadas aos cuidados dos pacientes. Outro profissional destaca que, embora dependa diretamente de uma enfermeira-líder, possui autonomia na prestação de cuidados e reporta necessidades individualmente ou em reuniões de grupo.

*Eu tinha autonomia nessas decisões no que diz respeito ao processo dos cuidados, no que diz respeito ao cuidado do doente e tudo com ele estava relacionado.* (EPo-07)

*No meu serviço, eu dependo diretamente de uma enfermeira, que é a enfermeira-chefe, que faz a gestão da unidade, e eu tenho níveis de autonomia em tudo que diz respeito à prestação dos cuidados, e é a ela que eu reporto as necessidades que vou sentido ou que vamos sentindo e, em muitas delas, o reporte é feito individualmente e em outras em reuniões de grupos que fazem frequentemente nos serviços.* (EPo-08)

Essas narrativas evidenciam que o PE proporciona aos enfermeiros uma estrutura que facilita a autonomia na prática assistencial, embora fatores como hierarquia e recursos financeiros possam influenciar o grau dessa autonomia. Compreender essas dinâmicas é essencial para fortalecer a prática profissional e a qualidade do cuidado prestado.

Quanto aos processos formativos relacionados ao modelo de gestão de enfermagem, salienta-se que desempenham um papel crucial no desenvolvimento da autonomia e liderança dos profissionais. As experiências compartilhadas por enfermeiros do Brasil, Espanha e Portugal oportunizam compreender melhor sobre como a formação e a prática influenciam suas competências gerenciais.

No Brasil, uma enfermeira relata a transição de uma postura centralizadora, vinculada ao modelo da administração científica, para uma abordagem mais democrática e participativa que oportunize a gestão da *performance* das trabalhadoras do campo da enfermagem. Ao delegar responsabilidades e preparar as enfermeiras administrativas para tomar decisões na sua ausência, ela assegura a continuidade da gestão e o desenvolvimento das habilidades de liderança de sua equipe. Essa prática não apenas facilita a gestão diária, mas também prepara a equipe para futuras transições e crises, oportunizando a sustentabilidade das operações.

*Eu comecei a desenvolver autonomia nas minhas enfermeiras, me dizem, então fui muito centralizadora,* [...] *gosto de ter controle das coisas, gosto de saber como vão as coisas, mas, ao mesmo tempo, gosto de dar autonomia para que tudo não fique centrado somente em mim, porque o que foi que eu aprendi é que aqui não é para sempre. Pode chegar o momento que eu tenha algum outro convite para alguma outra coisa ou que não seja mais interessante para a instituição me ter, meu cargo, e que venha outra pessoa e eu tenho que me preparar. Então, eu tenho enfermeiras administrativas; isso fez toda a diferença, nos finais de semana e feriados que eu deixava no hospital, então isso já foi excelente porque as meninas conseguem resolver quando eu não estou no hospital. Elas conseguem ter uma visão do que é uma gestão também, não só de enfermeira assistencial.* (EBr-06)

Na Espanha, um enfermeiro destaca a necessidade de aprimorar a formação contínua dos profissionais de saúde, especialmente daqueles em posições de gestão. Ele critica a estagnação de alguns supervisores que, após décadas na função, não se atualizam conforme as mudanças no ambiente hospitalar e nas práticas de saúde. Essa observação ressalta a importância da educação permanente e da adaptação às novas realidades do setor.

*Eu acho que a formação tem que ser melhorada para tudo que foi enviado pelo meio, e o mesmo que nós estamos aqui e temos preocupação com a vinda do aluno e não com os outros. Isso tem que ser para todas as pessoas que têm gestão. Não podemos ser supervisores 25 anos onde estamos vivendo na glória do passado, porque, há 25 anos, o hospital não era assim, nem a saúde nem nada.* (EEs-01)

Outro enfermeiro espanhol enfatiza a autonomia na gestão de leitos e na organização dos cuidados aos pacientes. Ele menciona a liberdade para decidir sobre exames, cuidados e agendamentos, bem como a gestão de consultas e visitas domiciliares. Essa autonomia é romantizada, contudo compreendemos que a autonomia regulada a protocolos institucionais que promovam fluxos mais efetivos pode possibilitar uma adaptação mais precisa às necessidades dos pacientes e uma gestão mais eficiente dos recursos disponíveis.

*Por exemplo, para gerir os leitos, para onde vai uma internação, para onde vai outra, a gente consegue isso, quando, por exemplo, decidimos certos exames ou certos cuidados, a que horas são feitos ou a que horas não são, dizer ao médico, bem, é melhor assim ou não é para organizar meu trabalho estou no hospital.* [...] *é totalmente autônomo, porque você tem sua consulta, você gerencia, você tem sua agenda,* [...] *quando você vai ver uma casa, você gerencia tudo lá.* (EEs-08)

Em Portugal, a formação de estudantes de enfermagem é enriquecida por programas de tutoria que visam desenvolver competências clínicas, teóricas e pedagógicas. Um enfermeiro português destaca a importância de os supervisores clínicos serem qualificados não apenas em conhecimentos técnicos, mas também em pedagogia, política, administração e comunicação, para melhor orientar estudantes e profissionais em desenvolvimento. Essa abordagem holística assegura que os futuros enfermeiros estejam bem preparados para os desafios da prática profissional.

*Papel de tutores ou de tutorias nos processos de formação de estudantes, seja no percurso de desenvolvimento inicial, seja nos percursos avançados. E, para isso, é necessário não só saber o ponto de vista da clínica, do ponto de vista teórico da enfermagem, é preciso saber também da pedagogia, da didática, de se comunicar, e o que nós pretendemos com essas formação do supervisor clínico era essencialmente que ajudassem esses enfermeiros a desenvolver competências nesta dimensão que eu referi, de se tornarem melhores, melhores comunicadores, de ficarem mais capacitados para comunicar, para ensinar os estudantes, outros profissionais que tivessem no curso de desenvolvimento avançados.* (EPo-02)

Outro enfermeiro português menciona a existência de um profissional responsável pela formação dentro da própria equipe que planeja e desenvolve qualificações conforme as necessidades identificadas. Essa prática promove o desenvolvimento contínuo da equipe, assegurando que as competências necessárias sejam constantemente atualizadas.

*Temos o enfermeiro responsável na própria equipe pela formação da equipe. Fazemos o planejamento e desenvolvemos essa formação. Obviamente, damos o conhecimento e pedimos diretamente ajuda em alguma situação pontual, mas temos a autonomia no sentido de identificar aquilo, quais são as necessidades da nossa equipe e promover esse desenvolvimento.* (EPo-04)

Essas narrativas evidenciam a importância de processos formativos contínuos e adaptativos no fortalecimento da gestão em enfermagem. A promoção da autonomia, a atualização constante, e a construção de habilidades de liderança e comunicação são essenciais para uma prática eficaz e alinhada às demandas contemporâneas da saúde.

## DISCUSSÃO

Nota-se que os hospitais dos três países estudados possuem características não uniformes nos cenários investigados, o que se explica por diferenças no modelo de gestão adotado e, consequentemente, no exercício da supervisão, bem como por fatores econômicos, políticos e sociais que interferem na gestão de cuidados e na construção/fortalecimento da autonomia profissional.

Destaca-se que a autonomia profissional do enfermeiro no ambiente hospitalar é um tema complexo, que vem se modificando ao longo do tempo, conforme o contexto histórico e social. Nota-se que, por influência do modelo biomédico vigente neste ambiente, há uma fragilidade da autonomia profissional associada à identidade profissional e à ideia de subserviência ao longo deste contexto histórico. A definição de autonomia profissional é considerada uma ideologia poderosa, que envolve a ideia de autoridade regulada para tomar decisões e liberdade de agir dentro do seu exercício profissional, associando experiência e conhecimentos para essa tomada de decisão. Além disso, possui *interface* à experiência do trabalho, através da participação na tomada de decisão e da capacidade de mudança nos processos de trabalho^(15)^.

Ainda nesse sentido, é válido trazer a concepção de autonomia do enfermeiro, permitindo que se pratique o mais alto nível de treinamento/saber científico para ofertar cuidados de saúde de qualidade, sendo valor significativo na satisfação no trabalho do enfermeiro^(7)^. Crenças baseadas no fazer médico confundem esse conceito na prática do enfermeiro e o seu objeto do cuidado. A concepção de autonomia no campo da educação traz a discussão do respeito aos direitos humanos e da ética no contexto do processo de ensino-aprendizagem e nas relações educativas^(8)^. É nesta perspectiva que esse pressuposto se assemelha à concepção da supervisão social, na qual entende a dimensão educativa da supervisão em busca do desenvolvimento do trabalhador como corresponsável na produção de cuidados em saúde^(10)^.

Corroborando este pensamento, destaca-se que os enfermeiros têm como objeto de trabalho o cuidado ao paciente, de modo holístico, na defesa da vida, sendo escopo de prática profissional. Ademais, em países como Portugal e Espanha, o modelo de gestão adotado evidencia fortes níveis de hierarquia na estrutura de poder organizacional que limitam a contribuição da supervisão para a construção/fortalecimento da autonomia. Isso ficou nítido durante a análise dos fragmentos de falas, os quais demonstram os níveis operacionais e o poder estabelecido no exercício da supervisão.

Estudo discutiu a autonomia e o trabalho significativo, tendo concluído que ambos estão positivamente relacionados ao nível de satisfação no trabalho para os enfermeiros, como condições de trabalho, reconhecimento e valorização profissional^(16)^. O entendimento sobre trabalho significativo está relacionado ao sentido do trabalho para a concepção de mundo do sujeito. Este estudo evidencia que o desenvolvimento da autonomia profissional demanda o alinhamento dos níveis de hierarquia da supervisão na estrutura de poder na perspectiva da gestão participativa. Reconhece-se a supervisão como instrumento gerencial que favorece o desenvolvimento do potencial da equipe de enfermagem e como estratégia que visa atender às necessidades de formação para a prática clínica, além de contribuir para construção/fortalecimento da autonomia profissional.

O processo decisório do enfermeiro divergiu nos três países. No Brasil, ele se mostrou alinhado com o modelo de gestão adotado e respaldado pelos instrumentos de gestão utilizados, incluindo-se o planejamento estratégico e a supervisão. Constatou-se, ainda, a presença da supervisão tradicional, bem como incentivo à gestão participativa, com estímulo à corresponsabilização dos atores envolvidos, em uma perspectiva de cogestão, características da supervisão social. Estudo analisa o desenvolvimento da autonomia por meio da liderança do enfermeiro e de condições atreladas à gestão participativa, em especial no que se refere à criação de condições para manutenção da força de trabalho de modo saudável^(17)^. Nesse sentido, a satisfação no trabalho é considerada elemento preditivo para permanência dessa força de trabalho na organização de saúde e, inclusive, na profissão.

Contudo, os resultados demonstraram o poder de um processo de tomada de decisão participativo focado no compartilhamento de conhecimento e poder, reconhecendo a inter e multidisciplinaridade como estratégias mais eficazes no modelo de tomada de decisão de racionalidade limitada, onde as decisões satisfatórias não são necessariamente as melhores.

A construção/fortalecimento da autonomia através da liderança é abordada em estudo que evidencia o surgimento de um novo tipo de líder, com características voltadas à busca do equilíbrio entre autonomia e responsabilidade, enfatizando o trabalho em equipe e a melhoria dos resultados assistenciais^(18)^. Embora não se tenha visualizado esta mesma preocupação em Portugal e Espanha, nota-se, nesses países, que o processo de decisão é controlado e, consequentemente, a autonomia é mais regulada. Nota-se a necessidade de direcionar esforços para fortalecer a supervisão clínica desde o processo formativo dos enfermeiros para fundamentar a oferta de cuidados de qualidade e seguro para os pacientes^(19)^. Neste contexto, a supervisão é considerada uma ferramenta gerencial para ajustamento entre a área operacional e a gestão estratégica, no intuito de oportunizar o alcance dos objetivos organizacionais.

Por sua vez, a produção de cuidados na perspectiva do PE guiado por uma teoria é uma potencialidade relacionada à contribuição da supervisão para construção/fortalecimento da autonomia do enfermeiro no ambiente hospitalar nos países ibero-americanos, o que sinaliza e reconhece o processo de enfermagem como um método na produção de cuidados, exigindo do enfermeiro conhecimento científico, julgamento clínico, pensamento crítico, compromisso com o próximo e responsabilidade na tomada de decisões. A autonomia do profissional de enfermagem deve ser consolidada mediante o uso da sistematização do cuidado de enfermagem. Isso inclui julgamento clínico e pensamento crítico, que embasam a tomada de decisão do enfermeiro, bem como a concepção da responsabilidade, sem restrições na colaboração profissional junto a outros profissionais de saúde, no âmbito do seu exercício profissional^(7)^.

Nesta mesma perspectiva, considera-se que a autonomia nos apresenta elementos constitutivos da compreensão da prática docente enquanto dimensão social da formação humana^(8)^, sendo um postulado importante para reflexão de ações que levam à fragilidade no acolhimento dos serviços de saúde. Esse pensamento possui relação com a temática da supervisão do enfermeiro defendida nesta pesquisa e entendendo a supervisão como uma prática social e que está implicada nos processos educativos^(10)^. Estudo constatou o reconhecimento da autonomia dos enfermeiros relacionada à implantação da Sistematização da Assistência de Enfermagem^(20)^. É considerada um caminho para construção/desenvolvimento/fortalecimento da autonomia para a profissão, por representar um modo de organização do cuidado de enfermagem reconhecida pelos enfermeiros, que permite a gestão do cuidado junto ao paciente, por exigir conhecimento científico, responsabilidade profissional e compromisso com o exercício profissional.

Ademais, os processos formativos relacionados ao modelo de gestão de enfermagem são uma potencialidade da supervisão que contribui para construção/fortalecimento da autonomia do enfermeiro no ambiente hospitalar em países ibero-americanos, que apresentam diferenças na forma como são conduzidos nos cenários investigados. Ainda nesta perspectiva, ressalta-se a importância da promoção de práticas educativas e da gestão do conhecimento nos serviços de saúde, do desenvolvimento de autonomia entre os membros da equipe e do estímulo ao crescimento organizacional, assim como o desenvolvimento de competências gerenciais para o exercício da supervisão do enfermeiro^(21)^.

Percebe-se, com base na análise dos fragmentos das falas selecionadas, sobretudo dos entrevistados de Portugal e Espanha, que os processos formativos são direcionados pelos erros ou não conformidades que ocorrem nas unidades de produção, características da supervisão tradicional. Tal constatação reitera a necessidade de proporcionar uma melhor formação profissional no acompanhamento de pessoas, cuidados, serviços e sistemas de saúde em prol de uma supervisão centrada no desenvolvimento organizacional e nas pessoas, atentando-se mais às metas dos Objetivos de Desenvolvimento Sustentável da agenda 2030. Entretanto, discute-se o processo formativo com a seguinte reflexão: “*É nesse sentido que reinsisto em que formar é muito mais do que puramente treinar*”^(8)^.

Ao analisar tal pensamento, observa-se uma preocupação nos serviços em ofertar cursos, qualificações em serviço e educação permanente previamente moldada baseados na necessidade do serviço ou problemas e casos reais. Entretanto, há necessidade de resgatar a curiosidade deste trabalhador, para que a busca pelo conhecimento seja constante. É nesta perspectiva que a supervisão social defende a dimensão educativa, tendo o trabalhador como protagonista desse processo.

A dimensão crítico-reflexiva, assim como a dimensão sócio-política, é esquecida no processo de trabalho da equipe de enfermagem, e isso tem impactos sérios na produção, sentido, qualidade e humanização do cuidado respeitoso. A dimensão política é essencial para valorização e reconhecimento das práticas em prol da qualidade de vida, dos projetos de felicidade e de uma sociedade harmônica e saudável.

Vale ressaltar que, no Brasil, foram identificados alguns aspectos relacionados ao processo formativo direcionado à preparação técnica e potencialidade da supervisão que contribuem e fortalecem a construção da autonomia no que se refere ao desenvolvimento do gerenciamento da unidade de produção e do processo de trabalho do enfermeiro no ambiente hospitalar. Estudo concluiu que, na última década, a autonomia do enfermeiro dentro do processo de trabalho em saúde vem se fortalecendo. Isso permite que ele atue nas práticas avançadas, visando não apenas aumentar o acesso de cuidados por parte dos usuários, como também mitigar os custos dos serviços de saúde^(22)^.

### Limitações do estudo

Este estudo apresenta algumas limitações que devem ser consideradas ao interpretar os resultados. Primeiramente, a pesquisa foi conduzida em hospitais de apenas três países ibero-americanos - Brasil, Espanha e Portugal -, o que pode não refletir a realidade de outras nações ou regiões com diferentes contextos socioeconômicos e culturais. Além disso, a seleção dos participantes foi limitada a enfermeiros que atuam em ambientes hospitalares específicos, o que pode não representar a diversidade de experiências e práticas existentes em outros contextos de saúde.

### Contribuições para as áreas da enfermagem, saúde ou políticas públicas

Este estudo oferece contribuições significativas para a área da enfermagem, especialmente no que diz respeito à compreensão das potencialidades e fragilidades da supervisão na construção e fortalecimento da autonomia dos enfermeiros em ambientes hospitalares. A pesquisa oportuniza compreender e refletir sobre práticas e modelos de gestão de enfermagem, em diferentes países ibero-americanos, que podem subsidiar a implementação de estratégias de supervisão mais eficazes e alinhadas às necessidades dos profissionais de enfermagem. Além disso, ao evidenciar a importância de processos formativos e do PE, o estudo reforça a necessidade de investimentos contínuos na educação permanente dos enfermeiros, visando aprimorar a qualidade do cuidado prestado e promover a autonomia profissional. Por fim, ao identificar limitações e fragilidades nos modelos atuais de supervisão, a pesquisa abre caminho para futuras investigações que possam aprofundar esses temas, contribuindo para o desenvolvimento de práticas de supervisão mais participativas e emancipadoras na enfermagem.

## CONSIDERAÇÕES FINAIS

Conclui-se que o estudo atingiu o objetivo proposto, que foi de analisar os contributos e atravessamentos da supervisão do enfermeiro, constatando uma prática social transformadora com implicações nos sistemas de cuidado à pessoa, família e coletividade, na busca do desenvolvimento do trabalhador como elemento de co-visão e de responsabilidade na produção de cuidados. Assim, este estudo possibilitou o melhor entendimento sobre o desenvolvimento da supervisão do enfermeiro na construção/fortalecimento da autonomia profissional no ambiente hospitalar em países ibero-americanos, com nuances específicas no campo das limitações e potencialidades, entendendo que o modelo de gestão adotado sofre influência de fatores culturais, sociais, econômicos e políticos.

## Data Availability

Os dados de pesquisa só estão disponíveis mediante solicitação.
